# 2543: Participatory development of a CTSA-wide Community Advisory Board: Enhancing community engagement at the Michigan Institute for Clinical & Health Research

**DOI:** 10.1017/cts.2017.193

**Published:** 2018-05-10

**Authors:** Jorge Delva, Adam Paberzs, Patricia Piechowski, Karen Calhoun, Diane Carr, Meghan Spiroff, Ayse Buyuktur, Kevin Weatherwax

**Affiliations:** University of Michigan School of Medicine, Ann Arbor, MI , USA

## Abstract

OBJECTIVES/SPECIFIC AIMS: To describe how Michigan Institute for Clinical & Health Research (MICHR) has engaged communities in its leadership and governance structure. This presentation will describe these practices, how they are being evaluated, and future plans for institute-wide engagement of communities in translational research. METHODS/STUDY POPULATION: Engaged partners from various communities across Michigan in various ways within MICHR’s Community Engagement Program. RESULTS/ANTICIPATED RESULTS: MICHR has utilized participatory practices in the development of the CAB to strengthen existing relationships and build new ones with potential partners. DISCUSSION/SIGNIFICANCE OF IMPACT: MICHR-wide Community Advisory Board (CAB) will ensure community voices are heard and utilized in leadership and strategic decisions for CTSA activities.

